# ASO Author Reflections: Fluorescence-Guided Sentinel Node Biopsy for Breast Cancer

**DOI:** 10.1245/s10434-020-09344-2

**Published:** 2020-11-13

**Authors:** Martha S. Kedrzycki, Daniel S. Elson, Daniel R. Leff

**Affiliations:** 1 Hamlyn Centre, Institute of Global Health Innovation, Imperial College London, London, UK; 2 Department of Surgery and Cancer, Imperial College London, London, UK; 3 Department of Breast Surgery, Imperial Healthcare Trust, London, UK

## Past

Sentinel lymph node biopsy (SLNB) in breast cancer is a mainstay of operative treatment in localized invasive disease. The procedure samples the first lymph nodes draining the tumor, which reflect the metastatic status of the axillary basin.^1^ The ‘gold standard’ technique utilizes radioisotope (RI) and blue dye (BD), however these agents are fraught with problems. RI necessitates hospitals follow Ionizing Radiation Medical Exposure Regulations for safe storage, use, and disposal.^2^ It is costly, requires special staffing for administration and monitoring of the patients, and necessitates an extra hospital visit for patients due to its pharmacokinetics.^3^ On the other hand, BD can elicit type 1 hypersensitivities, skin reactions, semi-permanent tattooing, and may fail to identify all sentinel nodes.^4^ Fluorescence-guided surgery using indocyanine green (ICG) has emerged as an alternate technique for sentinel node biopsy.

## Present

A meta-analysis was performed on a critical mass of high-quality research studies comparing fluorescence-guided SLNB and the dual technique. The results highlighted that ICG is comparable with the dual technique in SLN identification, and identifies 0.218 (*p* = 0.003) more SLNs per patient. Furthermore, the odds of identifying SLNs when using only ICG are similar to RI alone (odds ratio [OR] 2.58, confidence interval [CI] 0.35–19.08, *p* < *0.05),* but far superior to BD alone (OR 9.07, CI 6.73–12.23, *p* < *0.05*).^5^ The key findings of this paper are that fluorescence-guided SLN biopsy can also be used as an alternative to the gold-standard technique or RI alone, and should certainly be considered in centers that are unable to use RI. This has significant implications, with regard to both patient care and costs, given the streamlined patient journey, reduced adverse effects (lesser risk of allergic reactions and lack of skin tattooing), and diminished burden on hospital infrastructure, as well as decreased costs of the dyes and relevant equipment.^3^

## Future

Many questions still remain unanswered when it comes to ICG for SLN biopsy in breast cancer. Given that there have not been any randomized controlled trials comparing ICG and the dual technique, the data taken from these non-randomized studies may have been biased, and it is not possible to attribute certain outcomes (such as complications) to either technique in particular. Furthermore, no study has evaluated the learning curve of SLN biopsy using ICG, as surgeons in the meta-analysed studies were already proficient in the surgical procedure and any learning was of the novel technology. Future work should focus on addressing these queries in view of clinical adoption of ICG as a single agent for SLN biopsy.
